# Dynamic monitoring of public opinion on fertility intentions: based on the intersection of empirical and social media perspectives

**DOI:** 10.3389/fpubh.2026.1739460

**Published:** 2026-03-19

**Authors:** Xiaoning Wang, Jinyuan Fu, Jingru Fan, Jianglin Zeng

**Affiliations:** School of Data Science and Intelligent Media, Communication University of China, Beijing, China

**Keywords:** empirical analysis, population growth strategies, public fertility intentions, public fertility policies, text mining

## Abstract

**Introduction:**

In the context of China's rapid economic development and profound social changes, adjusting fertility policies has become a key national strategy. However, the long-term effectiveness of fertility incentives such as the three-child policy remains uncertain due to the lack of comprehensive economic and social support measures.

**Methods:**

This study innovatively utilizes Large Language Models (LLMs) to assist social media analysis, including intelligent noise removal and weighted sentiment analysis. By combining empirical data and a social media perspective, we developed the Dynamic Monitoring of Public Opinion on Fertility Intentions (DMPOFI). The aim of this paper is to analyze the factors influencing fertility intentions among Chinese women, providing theoretical support for policy formulation. The study analyzes 45,810 Weibo posts, all in Chinese, focusing on the public's views and sentiment toward fertility intentions. Through methods such as weighted sentiment analysis, LDA topic modeling, and TF-IDF, we gain insights into the changing public attitudes in recent years.

**Results:**

From the perspective of fertility intentions, the period of 2015–2016 was the most positive, while from 2017 to 2019, public attitudes toward fertility gradually became more rational and neutral. From 2020 to 2023, fertility intentions significantly declined, with neutral and negative emotions gradually dominating. The core factors influencing fertility intentions identified in this study include policy changes, social perceptions, economic pressures, work-life balance, cultural shifts, fluctuations in birth rates, and discussions on family planning.

**Discussion:**

Based on these findings, we propose a series of policy recommendations, urging the government to provide more flexible maternity leave policies, workplace support, and childcare services.

## 1 Introduction

Against the backdrop of rapid economic development and profound social structural changes in China, adjustments to fertility policies have become a pivotal issue in the national strategic agenda. As problems such as population aging and low birth rates intensify, these challenges pose severe threats to the high-quality and sustainable development of China's socio-economic system. Specifically, population aging not only leads to labor shortages and increased burdens on the older adults care system but also exerts far-reaching impacts on the country's economic development and social structure. Consequently, improving fertility willingness and formulating effective fertility policies have emerged as pressing issues that demand immediate attention.

However, understanding fertility intention is complex. In this study, we define fertility intentions not merely as the desired number of children, but as a multidimensional construct encompassing reproductive timing, planning, and the cognitive-emotional evaluation of childbearing behaviors (1). While existing literature provides a rich foundation for understanding these intentions, most empirical studies rely on traditional survey data (such as CGSS), which offer robust but static snapshots (2). In a society characterized by “social acceleration,” where policy shifts and public sentiment evolve rapidly (3), such methods often suffer from data lags, making it difficult to capture short-term fluctuations driven by immediate events (e.g., policy announcements or the pandemic) (4). To bridge this gap, our study shifts the analytical focus from “static points” to a “dynamic line,” aiming to capture the continuous trajectory of public sentiment (5).

This study combines traditional survey data with social media text analysis to capture real-time public sentiment and attitudes toward fertility issues (6). By integrating the continuous granularity of social media data (2015-2024) with the structural depth of survey data, we aim to achieve a more comprehensive understanding of the mechanisms underlying fertility intentions. Specifically, this study is anchored by a central research objective: to elucidate how dynamic public sentiments interact with structural factors to shape fertility intentions in the context of China's rapid social transformation. To systematically address this core inquiry, the analysis unfolds through three logical dimensions. First, we investigate the specific drivers of fertility intentions expressed in the digital sphere, ranging from economic pressures to emotional anxieties (7). Second, we assess the extent to which dynamic digital data can validate or challenge existing theoretical hypotheses regarding the divergence between policy incentives and individual intention (8). Finally, we explore how a real-time sentiment monitoring framework can enrich the theoretical understanding of fertility intentions and provide robust support for adaptive policy decision-making.

Addressing these questions is crucial for formulating and adjusting national population policies, especially given the intensifying aging problem and the continued decline in fertility rates in China. The primary contributions of this study are as follows:

- Establishing a Dynamic Monitoring Framework: We innovatively integrate traditional survey data with social media text data to construct a dual-methodological framework. Unlike single-source studies, this approach enables the cross-validation of “policy intent” versus “public sentiment,” providing a real-time mechanism to monitor the temporal dynamics of fertility intentions.
- Deepening Theoretical Integration: By incorporating Becker's Economic Theory of Fertility (9) and Social Acceleration Theory (3), we transcend the limitations of purely technical analysis. This study maps abstract economic concepts (such as opportunity costs) and temporal dynamics directly onto emotional patterns in social media, identifying the micro-foundations of the gap between policy incentives and fertility behaviors.
- Methodological Innovation in Social Science: We introduce LLMs for text preprocessing and weighted sentiment analysis to enhance the stability and accuracy of public opinion tracking. This demonstrates how computational social science methods can effectively complement traditional demographic inquiries, offering a scalable solution for understanding fertility behaviors amid rapid societal transformations (10).

## 2 Related work

In recent years, fertility intentions have attracted significant attention as an important variable affecting the structure of the working population and economic growth (11–14). Their formation is influenced by the interaction of multiple factors, including educational level, family economic status, gender differences, and regional disparities (15–18). Fertility intentions can be examined from three perspectives: social factors, family factors, and individual factors.

Social factors not only affect fertility intentions in specific regions, but also have a profound impact on overall fertility intentions (19–23). For example, maternity leave policies significantly influence male work-family conflict and are closely related to work and family characteristics (24). Policy changes are also important factors influencing fertility intentions. After the implementation of China's universal two-child policy, decisions about having a second child have shown greater dynamism and flexibility, often depending on negotiations between the couple (8, 19). In addition, the fertility intentions of the migrant populations are influenced by the social norms of their destination, and the immigrants tend to adjust their fertility behavior to suit the local residents (25).

At the family level, fertility intentions are constrained by family customs, economic status, and power structures (20). Although the influence of family roles has declined in post-industrial societies, family relationships still play an important role in shaping fertility decisions (25, 26). In rural areas, fertility attitudes have undergone a shift, especially with the weakening of gender discrimination (27, 28). Women of childbearing age living with their parents are more likely to have a second child (27).

Individual-level influences form the foundation of fertility intentions and should not be overlooked. Studies have shown that increased educational levels lead to a significant decrease in fertility rates among women in the United States (29). This inverse relationship aligns with Becker's Economic Theory of Fertility, which posits a quantity-quality trade-off. “As the opportunity cost of childbearing rises—particularly for educated women—parents tend to substitute the quantity of children with higher investment in the quality of each child” (9, 20). In the specific context of China, this theory helps explain why rising housing and education costs have become powerful contraceptives. Chinese scholars have provided profound insights into why fertility rates remain low despite policy relaxation. Beyond traditional economic constraints, emerging factors such as the frequency of internet usage have been shown to significantly influence fertility intention, often mediated by evolving gender role attitudes (6). Furthermore, empirical findings indicate that while policy relaxation released some accumulated demand, the sustained boost was limited by inadequate social support systems (30). Gender dynamics also play a pivotal role; the “motherhood penalty” in the labor market significantly reduces women's fertility intentions, as noted in recent sociological studies (31).

Overall, existing research suggests that fertility intentions is no longer a static demographic variable but a dynamic psychological response to external economic pressures and social uncertainties. This evolving complexity poses a challenge for traditional research methodologies: how can we capture these rapidly changing, anxiety-driven fertility considerations in real-time?

Empirical research combined with social media analysis provides a more comprehensive and dynamic perspective on fertility intentions. Traditional empirical research collects data through large-scale population surveys and questionnaires to analyze factors that influence fertility intentions (23, 32–35). For example, data from 36 countries have explored the influencing factors of fertility intentions (36). However, traditional empirical research has certain limitations. These studies are often static and periodic (37), requiring a long time to collect new data, making it difficult to reflect rapid changes in public sentiment and opinions in real time (38). This limitation is particularly acute when viewed through the lens of Rosa's Social Acceleration Theory. Rosa argues that modern society is characterized by a “temporal misalignment” where the pace of social change and event-driven anxiety outstrips the slower cycles of policy response and traditional academic inquiry (3). Consequently, static data fails to capture the “frenetic standstill” of public sentiment. This limitation is particularly acute when viewed through the lens of Rosa's Social Acceleration Theory. Rosa argues that modern society is characterized by a “temporal misalignment” where the pace of social change and event-driven anxiety outstrips the slower cycles of policy response and traditional academic inquiry (3). Consequently, static data fails to capture the “frenetic standstill” of public sentiment. With the popularity of social networks, the frequency with which the public expresses personal emotions and opinions on platforms has significantly increased (39, 40), and social media data, through natural language processing and sentiment analysis technologies, can dynamically reflect public attitudes toward fertility (41–43). Social media has been proven to be important in predicting public opinion dynamics and emotional transmission patterns (44). By using big data from the internet, it is possible to profile online users, extract basic information, uncover user preferences, and infer demographic characteristics (45). Empirical research and social media data analysis each have unique advantages. The former provides a solid theoretical foundation and comprehensive macro data, while the latter can dynamically and in real-time reflect public sentiment and views. Therefore, combining the two can more comprehensively capture changes in fertility intentions.

In domestic and international studies, descriptive statistics and logistic regression analysis are commonly used (16, 35, 46). For instance, research has explored the relationship between income inequality and variability in childbearing intention, and used Logistic regression to analyze its influencing factors (47). However, logistic regression is limited to focusing only on statistically significant factors, overlooking the interaction effects of multiple factors (48). Some studies have used CGSS data to perform ordered regression analysis to explore the impact of public spending on individual fertility intentions (49).

With the development of machine learning algorithms, researchers have begun to adopt these advanced technologies to analyze fertility intentions (50). Researchers have improved the accuracy of sentiment prediction using topic models, text summarization, and TransModality methods, which integrate multimodal information, such as text, visual, and acoustic data (7, 51).

This paper is based on data from 2015 to 2024 and explores the trends in fertility intentions over the past decade, while delving into the multiple underlying influencing factors. Through a review of the literature and theoretical analysis, the study identifies negative factors that may suppress fertility intentions—such as economic burdens, childcare costs, and work pressures—as well as positive factors that could enhance fertility intentions, including policy support and social welfare. Building on this foundation, empirical methods are employed to examine the roles of these factors across different time periods. The findings are expected to provide both a theoretical basis and practical guidance for optimizing fertility policies and improving family support systems.

Compared to previous studies, this paper more comprehensively combines both social media analysis and empirical verification analysis. It utilizes various classic sentiment analysis and social media mining techniques, along with LLMs, to dynamically explore the changes in fertility willingness and the influencing factors from 2015 to 2024. Crucially, this dual approach allows us to empirically validate these theoretical frameworks in the digital age: using social media text to trace the “economic calculations” described by Becker, and establishing a dynamic monitoring system to address the “temporal lags” identified by Rosa.

## 3 Materials and methods

The purpose of this study is to analyze the multi-dimensional factors that affect public fertility intentions. In particular, we focus on how policy changes influence fertility intentions and its expression in emotions and attitudes. Through comparative analysis of empirical data and social media data, we reveal the trends in public fertility intentions over time and systematically extract the factors influencing these intentions.

### 3.1 Materials

#### 3.1.1 Data sources and pre-processing

Data for this study were collected using the Octopus web scraping tool and custom Python scripts from the Weibo platform. We selected Weibo as the data source primarily because it is one of China's most influential public-opinion platforms, characterized by a large user base, open topics, and strong interactivity, making it effective for capturing dynamic public discussions on fertility issues. Based on the latest user report released by Weibo and relevant academic literature (52), its user base skews young, urbanized, and includes 55% female users, which may introduce demographic biases. However, this profile aligns closely with the core demographic discussing fertility issues in China (53). Additionally, all data collection processes were performed in accordance with Weibo's privacy policies and ethical guidelines (54), ensuring that only publicly visible posts were gathered. Personal information, such as usernames, profiles, or specific locations, was excluded from the analysis to maintain user privacy and anonymity. The dataset focuses on topics related to fertility. A total of 104,420 posts were collected, providing a comprehensive reflection of user discussions and views on fertility policies and intentions. To ensure data completeness and accuracy, efficient strategies were employed during the data collection process. Specifically, keywords such as “fertility intentions,” “fertility policy,” “second child,” and “third child” were used to retrieve relevant Weibo topic links. The data covers the period from 2015 to 2024, with data for each month processed separately. The scraped fields are listed in Table 1.

**Table 1:** Table of data field descriptions.

**Data field**	**Data type**	**Description**
Poster	String	User's name who posted the content
Post time	Datetime	The time the post was published
Post link	String	Unique link to locate the post
Content	Text	Main content of the post
Reposts	Integer	Number of reposts showing reach
Comments	Integer	Number of comments showing interaction
Likes	Integer	Number of likes showing approval
Keywords	String	Keywords for topic analysis

For the validation analysis of CGSS, the datasets publicly available for both the 2021 and 2023 waves were used. The CGSS surveys cover 31 provincial-level administrative regions (excluding Hong Kong, Macau, and Taiwan) and provide nationally representative samples. The survey collects data on various socio-economic characteristics and family information. Data preprocessing included the removal of duplicates, noise reduction in text using a large language model, elimination of outliers, and correction of time values. See supporting materials for details.

#### 3.1.2 Data analysis and visualization

**Figure 1:**
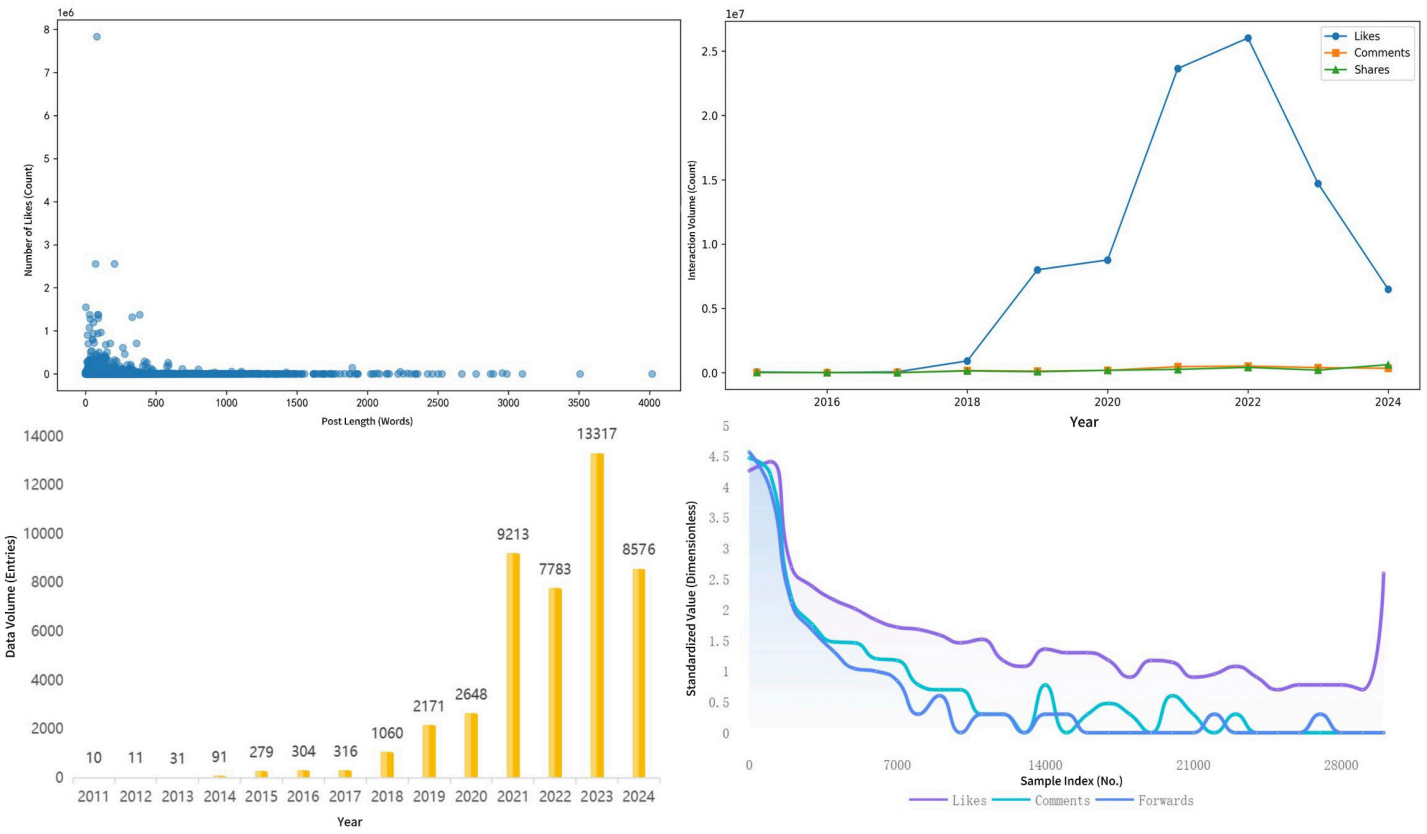
Comprehensive descriptive analysis. **(Top-left)** Histogram of the annual distribution of the crawled data; **(top-right)** line chart of likes, comments, and shares over the years; **(bottom-left)** scatter plot of likes vs. post length; **(bottom-right)** logarithmic distribution line chart of the number of likes, comments, and shares in different intervals.

After data preprocessing, a total of 45,810 Weibo posts were retained. To further explore the overall distribution of these data, a descriptive analysis was conducted. Figure 1 presents the visualization results of the descriptive analysis, including the distribution of posts over time, the relationship between user engagement (likes, comments, shares) and post length, and the distribution of engagement both in terms of quantity and time.

The annual distribution analysis reveals the trends in public discussions on fertility issues over time. Notably, 2023 had the highest volume of data, with 13,317 posts, accounting for 29.16% of the total, followed by 2021 and 2024, which accounted for 20.18% and 18.78%, respectively. This indicates a significant increase in public attention to fertility issues in recent years, particularly after 2021, likely driven by factors such as social policies, economic pressures, and demographic changes. Discussion activity was also notably higher in 2022, with 7,783 posts, accounting for 17.04%. Data from 2014 to 2022 were found to be too sparse and were excluded from further analysis. Therefore, this study focuses on the dynamic changes in fertility intentions between 2015 and 2024 (January–October). Additionally, post engagement follows a typical long-tail distribution, where most posts have low interaction rates, but posts with higher interaction rates, though fewer, still exist. It is evident that 2022 marked a key turning point, as the number of likes surged, likely related to the large post volume and the increase in active internet users during this period.

In addition to the descriptive analysis, this study also performed named entity recognition (NER) and part-of-speech (POS) tagging, which provided the foundation for subsequent analyses.

### 3.2 Methods

In this study, all text analysis models and tools used were specifically chosen to ensure they could effectively process Chinese language data. Since the Weibo posts used in this research are in Chinese, we made sure to utilize tools optimized for the Chinese language, including SnowNLP for sentiment analysis and GPT-4o-mini for understanding text nuances. All subsequent analysis methods, including LDA topic modeling, word embedding, and TF-IDF keyword extraction, were applied directly to the original Chinese text.

#### 3.2.1 Empirical analysis of sentiment analysis using LLMs and related methods

A series of advanced text analysis methods were selected for this study, which together constitute a comprehensive and in-depth research framework. In terms of model selection for sentiment analysis, SnowNLP, a dictionary-based tool, is widely used for Chinese sentiment analysis due to its stability and reproducibility in past research (55–57), making it a reliable tool for generating baseline sentiment scores. However, given the nuanced and context-dependent nature of discussions on fertility intentions, especially those involving policy and personal emotions, we incorporated ChatGPT-4o-mini (58–60), a large language model that excels in understanding subtle semantic differences and contextual dependencies in text. This combined approach allows us to benefit from the stability of traditional methods while leveraging the advanced capabilities of LLMs for deeper semantic understanding, enhancing the overall accuracy of sentiment classification (61–63). Secondly, the LDA topic model can automatically discover hidden topics in a document, revealing the “story line” behind the document, i.e., the topic discussed in the document and its importance. This is crucial for extracting valuable information from a large number of social media posts. Word co-occurrence matrix and semantic network analysis help us to understand and analyze the relationships between words, revealing the inner structure and semantic relationships of knowledge, which is useful for understanding the multidimensional nature of complex topics such as fertility intentions. The TF-IDF method filters out common words and retains important words by evaluating word frequency and inverse document frequency, which is crucial for generating representative word cloud maps and understanding key information in text. The K-Means algorithm combined with word vector embedding technology is able to convert textual data into numerical form for effective cluster analysis. This is useful for dealing with massive text data and discovering patterns in the data. Finally, the t-SNE method is a technique that integrates dimension reduction and visualization, which can effectively map high-dimensional data into two- or three-dimensional space for intuitive visualization, which is very important for understanding the clustering effect and data structure. In addition to this, the validation analysis uses Logistic binary regression to further verify the validity of the previously mined influencing factors.

To test the validity of existing theories and hypotheses, we conducted an empirical analysis using CGSS data to verify the robustness of the conclusions drawn from text analysis. When the results from CGSS data analysis align with those from text analysis, it enhances the reliability of the findings.

Data Selection: Variables were selected based on their theoretical relevance to fertility research. A37 measures the desired number of children and serves as the core indicator of fertility intention. A1 reflects household size and family structure, while A64 captures economic conditions that may influence childbearing decisions. A47 measures attitudes toward governmental fertility policies, and A2 provides key demographic information on gender. Together, these variables offer a comprehensive framework for examining determinants of fertility intentions across both the 2021 and 2023 CGSS waves.

Data Preprocessing: As shown, standard preprocessing steps were conducted, including removal of duplicate cases, cleaning of textual responses, elimination of outliers, and correction of inconsistent values. Descriptive statistics for the processed dataset are presented in Table 2.

**Table 2:** Frequency and percentage of willingness to give birth.

**Dependent variable**	**Option**	**Frequency**	**Percentage (%)**
Willingness to give birth	Yes	7,215	96.251
	No	281	3.749
	Sum	7,496	100

## 4 Results

### 4.1 Combined with the sentiment analysis of large language model and the empirical analysis of related methods

In this study, the posts related to fertility intentions on social media from 2015 to 2024 will be dynamically analyzed to reveal the changes in public attitudes during this period. A multi-level sentiment analysis approach will be adopted. First, the SnowNLP tool will be used for preliminary sentiment assessment to obtain basic sentiment scores (plain_score). Additionally, an advanced LLM, GPT-4o-mini, will be introduced to capture a more precise fertility intention score (relation_score) expressed in the user-generated texts. This combination of traditional and modern technologies will contribute to a more comprehensive understanding of the subtle shifts in public sentiment. Ultimately, a weighted summation approach will be employed to calculate a composite sentiment score, as described by the following formula:


$$\begin{array}{l} score=\text{plain\_score}\times 0.3+\text{relation\_score}\times 0.7 \end{array}$$ (1)

The weighting scheme is informed by commonly adopted practices in prior hybrid and ensemble-based sentiment analysis studies rather than being arbitrarily defined. Existing literature frequently employs moderate asymmetric combinations—such as 0.5/0.5, 0.4/0.6, 0.7/0.3, or 0.8/0.2—when integrating lexicon-based methods with context-aware models (71–74). Within this established range, we assign a lower weight (0.3) to the SnowNLP score to retain a stable and reproducible sentiment baseline, and a higher weight (0.7) to the GPT-4o-mini score to better capture context-dependent and nuanced emotional expressions. This configuration represents an intermediate and empirically grounded choice, balancing methodological robustness and semantic sensitivity (75, 76). Moreover, in empirical studies that integrate fertility economics and social theories to examine fertility intentions, similar weighting strategies have been adopted. Such studies often employ hybrid or ensemble modeling approaches with asymmetric weight allocations, retaining a stable analytical baseline while assigning greater weight to context-aware models (17, 77).

**Figure 2:**
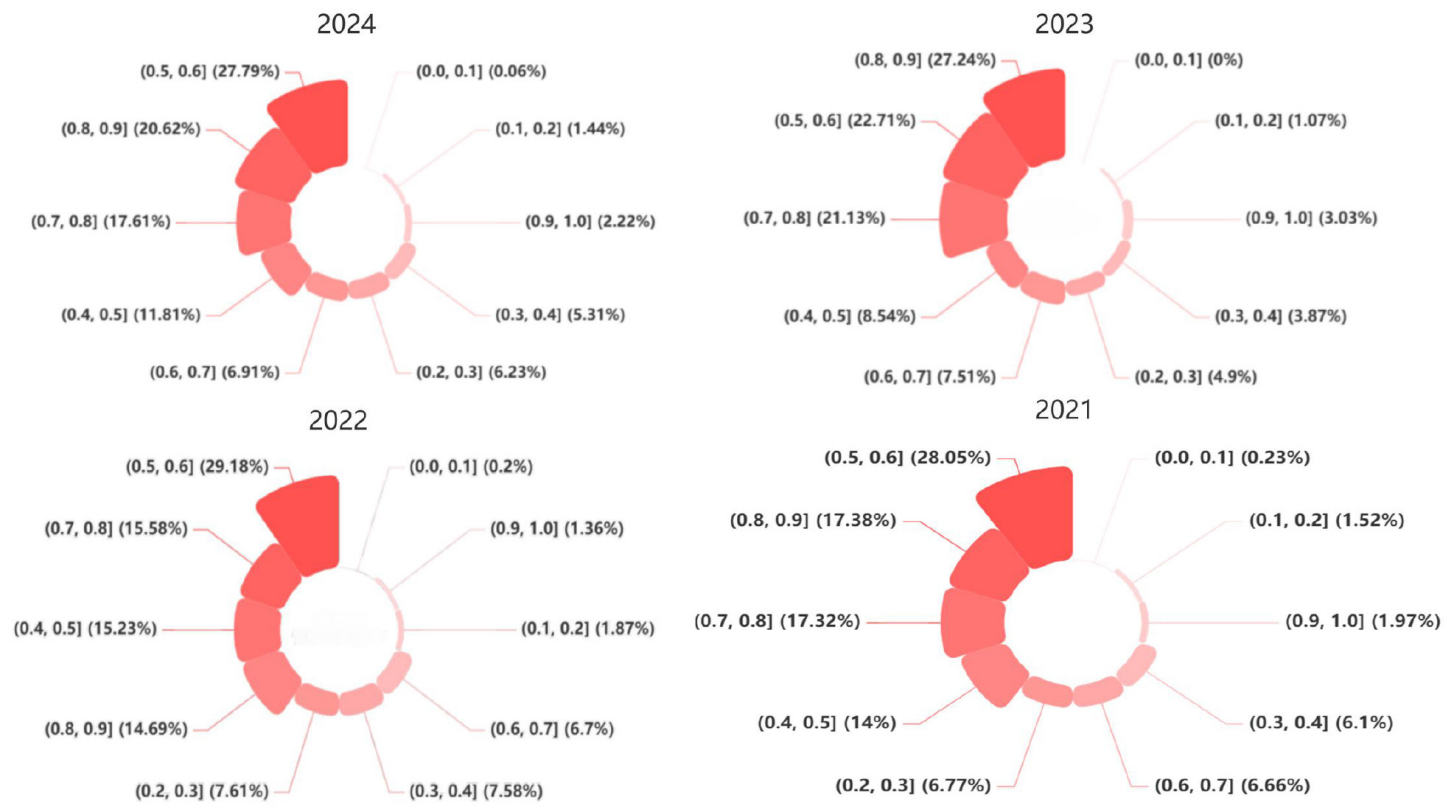
2021–2024 sentiment attitude interval proportions nightingale rose diagram.

Schematic diagram of some text emotion analysis results are shown in Table 3. This comprehensive score will help this study more clearly understand the public's attitude toward fertility and divide it into the following ten levels. Next, a Nightingale rose diagram will be plotted to visually display the proportion of sentiment attitude intervals for each year from 2015 to 2024, illustrating the changes in public sentiment toward fertility intentions over time. A comparative display of the sentiment attitude interval proportions for the past three years will be shown in the rose diagrams for 2022, 2023, and 2024, as depicted in Figure 2. The 2015 to 2024 sentiment Analysis Summary table is shown in Table 4, when conducting an in-depth analysis of fertility intentions between 2015 and 2024, the period can be divided into several key stages to better understand the changes in public emotional attitudes and the deep reasons behind them.

**Table 3:** Schematic table of the results of the sentiment analysis of some texts.

**Body of the post**	**Plain_score**	**Relation_score**	**Score**
Leung says China may face the world's worst low fertility crisis Since population expert Leung is so concerned about population issues, dare he investigate a little deeper?...	0.998253	0.3726	0.560296

**Table 4:** Summary table of annual emotion analysis from 2015 to 2024.

**Year**	**Sentiment analysis results**
2015	More positive than 50 per cent to 57 per cent and very positive to almost 3 per cent
2016	More than 9 per cent were very positive, and more than 67 per cent were more positive
2017	Positive performance reaches 46 per cent, with less than 10 per cent of the negative major categories combined
2018	Positive and neutral are similar at around 22 per cent.
2019	Performance positive and performance neutral are similar, with a small increase in Performance Positive compared to last year.
2020	For the first time, performance neutral exceeds Performance Positive at 25 per cent, with the latter at 23 per cent.
2021	Compared to the previous year, performance neutrality rose further to 28 per cent, while the total positive category share was 35 per cent, with less than 2 per cent being very positive.
2022	The share of performance neutrality continued to rise to 28 per cent, with the more positive exceeding the positive for the first time
2023	Compared to the previous year, the proportion of neutral performers rose further to 29 per cent, with under-neutral outpacing more positive, and the total of neutral categories at 51 per cent.
2024	The combined share of the positive category, at 40 per cent, rebounded, but remained below the share of the neutral category

First, the period from 2015 to 2017 can be regarded as the discussion period of the two-child policy. At this stage, public sentiment toward fertility is generally positive, characterized by relatively optimistic emotional expressions. From 2018 to 2019, the characteristics of the fertility policy reflection period are more and more obvious. The significant decline in the number of births in 2018 has triggered widespread social concern and reflection, and public discussions increasingly shifted toward concerns and reflections on fertility-related issues. From 2020 to 2023, the implementation of the three-child policy marks a new policy transition period. At this stage, public sentiment tends to be cautious, with the proportion of neutral people for the first time outpacing the proportion of positive people, indicating a more cautious and deliberative emotional stance when examined alongside contemporaneous topic modeling and semantic analysis results. Finally, 2024 has become an important node in the relationship between economic development and fertility intentions. At this point, positive emotion rebounded, although it was still lower than the proportion of neutral emotion.

Taken together with contemporaneous topic modeling and semantic analyses, these sentiment patterns provide a macro-level context for understanding how public discussions and thematic structures evolve across different policy periods.

By constructing a theme-topic co-word matrix, the paper analyzes the number of co-occurrences between topics. On the whole, policy subsidies and workplace balance are the two core topics discussed, and they are closely related to other topics, especially the gender perspective, the three-child policy and the birth rate are the most significant impacts, which indicates that financial support and workplace support are the two most important aspects when the society discusses how to increase the birth rate and implement the three-child policy. The high co-present value of gender perspective and workplace balance indicates that gender equality cannot be ignored in population policy and fertility related discussions, especially the balance between women's workplace and family responsibilities, which is an important consideration in driving policy formulation; Although fertility encouragement is a direct measure to increase the birth rate, its low co-present value with other topics indicates that incentive policies alone are not enough to solve the problem, and more comprehensive measures such as economic support and workplace balance are needed. At the same time, this study constructs the co-word matrix for each year and the whole keywords, see supporting materials for details.

Synthesizing the LDA results and the semantic network analysis, the findings indicate a progressive shift in public discussions from policy-oriented expectations to structurally embedded concerns, particularly those related to economic pressure, workplace balance, and gendered responsibilities. While specific policy terms remain visible, the discussion increasingly centers on everyday constraints shaping fertility decisions, suggesting a transition from policy awareness to lived-experience-driven discourse.

The themes and keywords for the Weibo posts from 2015 to 2024 will be extracted annually, as shown in Supplementary material. Through the visualization analysis of the LDA results, the topics for each year were categorized based on their commonalities. It was found that: the first category includes the years 2015–2017; the second category includes 2019, 2020, and 2022; the third category includes 2018, 2021, and 2024; and the fourth category includes 2023. The theme-keyword extraction results for the posts from 2015 to 2024 include the following core topics: Three Child Policy, Differences in Gender Perspective, Policy Subsidies, Fertility Incentives, Marriage and Childbirth Choice, Workplace Balance, Birth Rate, and Fertility Planning.

Firstly, the analysis starts from the overall semantic network. From the Supplementary material, it can be seen that the center of the network revolves around the words “fertility”, “life”, “family”, “society”, “choice” and so on. These nodes have many connections in the figure, indicating that they are the core topic of the entire network. This means that, on the whole, issues related to fertility, lifestyle, family and society dominate the discussion. The period from 2015 to 2024 is divided into four stages by the analysis of the co-word matrix. Then, representative graphs of each stage will be selected for analysis.

According to the 2016 graph in the upper left corner of the Supplementary material, it can be seen that 2016 was dominated by discussions about newborns, mothers, and related topics. The“newborn” in the figure is the core node of the network, connecting a large number of other nodes, indicating that the discussion around newborns was the most important topic in 2016. The discussion involved births, babies, children, etc. The other major node closely linked to “newborn” is “child”. This suggests that the discussion is not limited to the newborn stage, but extends to the subsequent stages of parenting, involving the interaction of parent and child.

According to the 2018 graph in the upper right corner of the Supplementary material, it can be seen that the nodes and connections are more dense, reflecting a broader and deeper discussion. This chart is significantly more complex than before, indicating that the topics discussed in 2018 cover more aspects and are more related to each other. Although there is no obvious single core node in the diagram, it can still be seen that there are several relatively larger nodes. For example, fertility policy, family responsibilities, economic impact and other issues. 2018 is the late stage of the full implementation of the two-child policy. With the implementation of the policy, the attention of the society on fertility is no longer limited to the policy itself, but more is discussed in combination with multiple factors such as social economy and family responsibilities.

The gradual development from the single-topic core (such as newborns and mothers) in 2016 to the multi-topic interaction in 2018 reflects the gradual prominence of the complexity of fertility issues in society. More and more factors are being incorporated into the discussion of fertility issues, forming a complex network of social, economic, cultural and policy interweaving. The 2020 graph in the lower left corner of the Supplementary material shows fertility related words in the center of the network graph. Although the three-child policy will not be officially implemented until 2021, there are already discussions about a three-child policy in 2020. Words such as “three children”, “liberalization” and “policy” in the picture show social speculation and discussion that the policy may be further relaxed. Words related to economic and social pressure, such as “economy”, “society”, “cost”, “work”, “pressure” are linked to “fertility”, “marriage”, “family”, etc, suggesting that economic pressure and social responsibility are influencing people's reproductive choices. At the same time, the trend toward unmarried infertility and policy changes (e.g., subsidies, recommendations) reflect the complexity and diversity of fertility issues. The social discussion on women's reproductive choice is further deepening, and economic pressure and childcare burden are still important factors affecting fertility.

By calculating the TF-IDF values, important keywords were identified, providing certain support for clustering.

**Figure 3:**
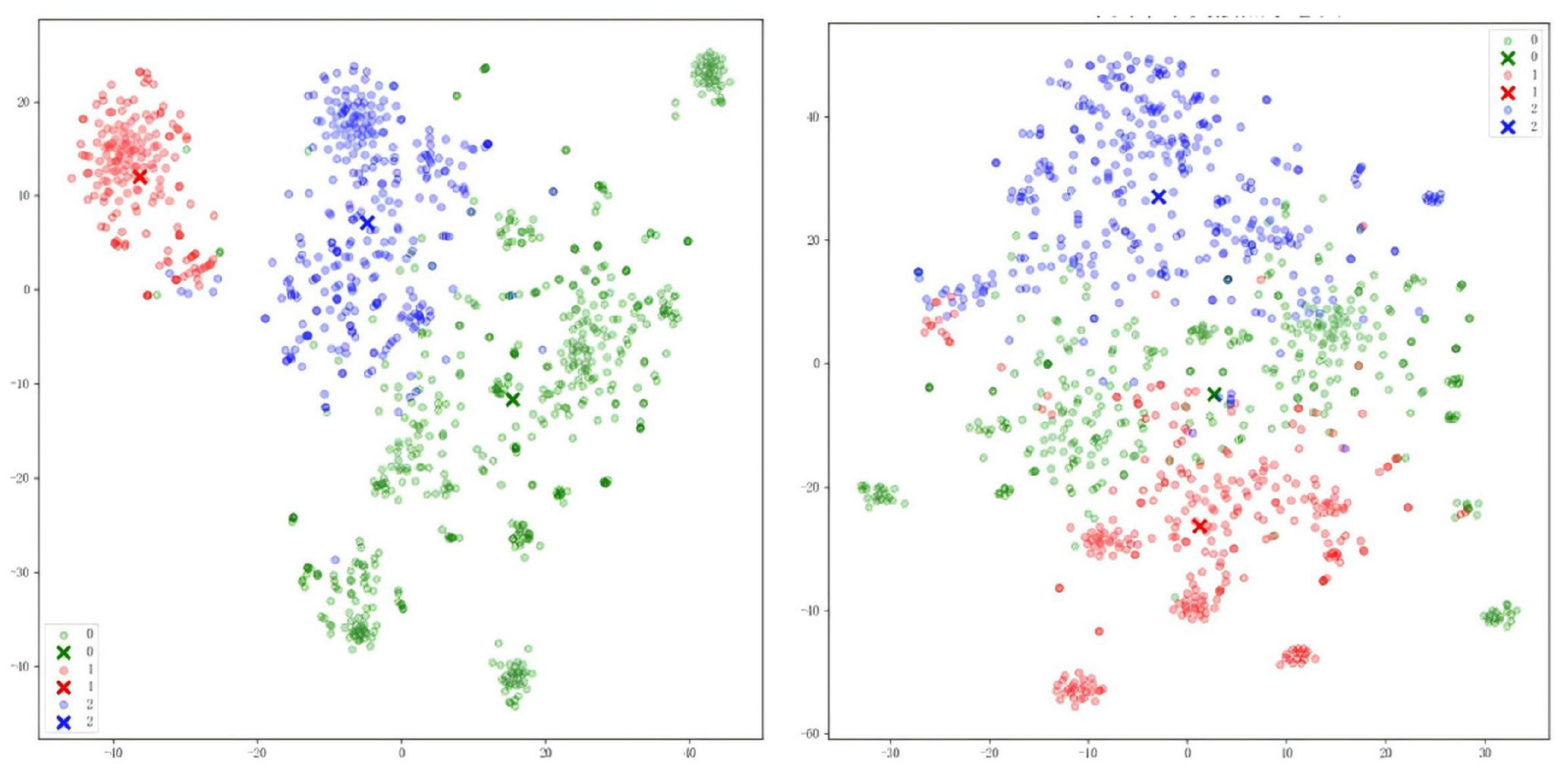
Schematic diagram of text clustering scattered points in 2023 and 2024.

In addition, to perform effective clustering analysis of the text, this study introduced word embedding techniques to extract text features. The word embedding model used here is OpenAI's high-performance, low-cost, multilingual-supported text-embedding-3-small, which supports a maximum context input of 8,192 tokens and outputs up to 1,536 vector dimensions. After converting the text information into numerical form, clustering algorithms can be applied to classify the text. In this study, the K-Means clustering algorithm was chosen, forming three clusters based on the post content, coded as 0, 1, and 2. The t-SNE method was then used for dimensionality reduction, mapping the 1,536-dimensional word vectors to a two-dimensional space, which was subsequently visualized and represented in a scatter plot. Figure 3 shows the clustering results for 2023 and 2024.

After word embedding processing, K-means clustering, and t-SNE dimensionality reduction, the text data were clearly divided into three categories based on color. According to the scatter plot, it can generally be concluded that the clustering separation is good. Next, corresponding explanations were provided for each cluster. Due to the large volume of text data within the same cluster, it was not possible to effectively extract and summarize the internal relationships of different clusters. Therefore, this study utilized a large language model to summarize the clusters, analyze the text, and generate the topics for each cluster to help understand the characteristics of each cluster. The results of text clustering on the topic of Weibo reproductive intention in 2024 are shown in Table 5. The following Logistic verification analysis will analyze the influence of different levels on the public's reproductive intention together with the clustering analysis of this module.

**Table 5:** Comprehensive table of cluster analysis and logistic validation analysis results.

**Clustering results**	**Commentary text topics**						
The tension between individual choice and social expectations	The importance of fertility, family and personal choices, and the resulting social implications and responsibilities are explored in different perspectives.						
Fertility policy and demographic concerns	In the current social environment, the issue of childbearing is not just a matter of personal choice, but is influenced by multiple factors such as economic, cultural and social structures.						
Limitations of maternity allowances and fundamental needs	Discussions centered around mothers' postnatal and childbirth-related experiences and feelings. The comments express mothers' deep emotions toward their newborns, share their physical conditions and experiences of childbirth, record their feelings of labor and reflect on postnatal changes.						
**Logistic analysis results**	**Regression coefficient**	**Standard error**	**Wald**	***P***	**OR**	**OR 95%CI (lower)**	**OR 95%CI (upper)**
Constant	–4.255	0.346	151.165	0.000^***^	0.014	0.007	0.028
Economic situation	0.147	0.080	3.356	0.067^*^	1.158	0.990	1.356
Family size–family structure	–0.237	0.040	34.475	0.000^***^	0.789	0.729	0.854
Government intervention	0.286	0.048	34.834	0.000^***^	1.331	1.210	1.463
Distinguishing between the sexes	0.151	0.124	1.472	0.225	1.163	0.911	1.484

^***^, ^*^ represent 1 per cent and 10 per cent significance levels, respectively.

### 4.2 Logistic regression results

According to the validation analysis results presented in Table 5, the determinants of fertility intentions exhibit both stable patterns and meaningful shifts between the 2021 and 2023 CGSS datasets. Economic situation does not reach statistical significance in either year (2021: *p* = 0.067; 2023: *p* = 0.646), indicating that self-perceived household economic conditions are not a decisive predictor of fertility intentions in the two survey waves. Gender is similarly insignificant in 2021 but becomes significant and negative in 2023 (*p* < 0.001), suggesting that women in the more recent survey are less likely to express fertility intentions—a trend consistent with the growing visibility of maternal burdens in public discourse.

Family size is significant in both years, though with opposite coefficient signs. In 2021, larger households are associated with lower fertility intentions, whereas in 2023 the relationship becomes positive. This shift may reflect changing interpretations of family support and resource-sharing under the evolving policy environment after the implementation of the three-child policy. Government intervention remains highly significant in both years but also shows a reversal in effect direction. In 2021, stronger agreement with limiting government involvement corresponds to lower fertility intentions; by contrast, in 2023 it is associated with higher fertility intentions. This pattern suggests that public attitudes toward state intervention have diversified, with individuals increasingly distinguishing between policy pressure and personal agency in reproductive decision-making.

These quantitative findings align with the clustering results, particularly the themes of personal choice, policy concerns, and maternal experiences. Consistent with the regression outcomes, economic topics did not emerge as central in the text-based analyses, while issues related to motherhood and autonomy appeared more prominently.

Taken together, these findings suggest that individuals may distinguish between expressing general support for fertility-related discussions and making concrete fertility decisions. While many respondents acknowledge fertility issues and policy debates, they may prioritize personal autonomy and family preferences, resulting in stable fertility intentions that are less responsive to policy signals alone.

## 5 Discussion

It is critical to clarify that the fertility-related sentiments and attitudes analyzed throughout this discussion are derived exclusively from social media discourse, and thus cannot be directly conflated with the actual fertility intentions or realized fertility behaviors of the general population. The findings of this study are bounded by the characteristics of online expressions rather than offline behavioral decisions. However, precisely by leveraging these unique digital characteristics, this study innovatively introduces the “temporal misalignment” mechanism. This framework reveals how the lag between high-frequency fluctuations in public sentiment and low-frequency policy adjustments contributes to the decline in fertility intentions, thereby breaking through the limitations of traditional static research.

### 5.1 From the perspective of fertility intentions: temporal dynamics and shock responses

Our longitudinal sentiment analysis, synthesized with major socio-political events, reveals distinct phases in public fertility-related sentiment. From 2015 to 2016, sentiment peaked, driven by the “institutional release” effect of the universal “two-child policy.” This policy shift directly stimulated reproductive enthusiasm, aligning with findings that policy relaxation can temporarily unlock suppressed demand (30). However, from 2017 to 2019, sentiment stabilized but began a downward trajectory. Despite policy incentives, the rising direct costs of housing, education, and healthcare exerted a crowding-out effect. This pattern is consistent with Becker's (9) “Quantity-Quality Trade-off” framework, where the rising “shadow price” of childbearing compels families to substitute the quantity of children for higher quality investment, thereby suppressing fertility intentions (64).

From 2020 to 2023, fertility-related sentiment experienced a precipitous decline. The exogenous shock of the COVID-19 pandemic amplified economic uncertainty. This rapid shift from optimism to neutrality/negativity reflects the phenomenon of “Social Acceleration” (3): high-frequency public anxiety outpaces the slower cadence of policy adjustments, creating a “temporal misalignment” that fosters a wait-and-see attitude. By 2024, as macro-conditions improved and policies were refined, a slight recovery was observed. Yet, the dominance of neutral sentiment suggests that restoring confidence requires addressing deep-seated structural rigidities rather than relying solely on post-crisis recovery (65).

The divergence between digital expression and empirical survey results reveals a mechanism of “temporal misalignment” explained by Social Acceleration theory. Topic modeling of social media data captures high-frequency emotional fluctuations (e.g., anxiety about the pandemic) that react instantaneously to external shocks, whereas regression analysis based on survey data captures the slower, more rigid adjustments of fertility intentions. This lag creates a window where policy interventions may arrive too late to reverse already-formed negative sentiments.

### 5.2 From the perspective of influencing factors: structural constraints and value shifts

Through LDA topic modeling and co-occurrence network analysis, this study identified several core determinants of fertility intentions, including policy dynamics, social norms, economic pressure, and work-life balance.

First, the transition from the two-child to the three-child policy generated significant discourse, yet public attention increasingly scrutinized the “motherhood penalty” (31). Discussions on women's reproductive rights and the unequal distribution of care labor highlight the “incomplete gender revolution” (66), where gender equity in the public sphere (work) has not been matched by equity in the private sphere (home).

Economically, factors such as dowries, income, and housing costs emerged as pivotal. However, contrasting the high volume of economic complaints on social media with demographic realities suggests a gap between emotional venting and rational decision-making. This supports the “low-fertility trap” hypothesis (67), where economic pressure triggers negative sentiment, while actual behavior is constrained by the discrepancy between “ideal parity” and “actual intention” (68). Furthermore, the strong semantic nexus between “workplace balance” and “gender perspective” underscores that fertility decisions are intrinsically linked to labor market fairness. Finally, the rising visibility of the “DINK” (Double Income, No Kids) lifestyle and the delay in marriage reflect the “Second Demographic Transition” (SDT) (69), indicating a profound shift toward individualism and self-actualization that transcends purely economic calculations.

### 5.3 Policy implications for enhancing fertility intentions

To translate the insights from our dynamic sentiment analysis into actionable policy, we propose a shift toward Sentiment-Responsive Governance. This approach uses real-time public sentiment as a feedback mechanism to design more agile and effective interventions. Based on our core findings—particularly the centrality of economic pressure and workplace balance in public discourse and the identified “temporal misalignment” between policy announcement and public reception—we offer the following evidence-based recommendations.

First, address the structural economic constraints directly linked to negative sentiment. Our analysis confirms that concerns over childcare costs, housing, and education dominate fertility-related anxiety, aligning with Becker's (9) Quantity-Quality Trade-off framework where rising “shadow prices” suppress fertility. Therefore, merely relaxing policy limits is insufficient. We recommend long-term, targeted financial subsidies in education, healthcare, and housing to lower the direct and opportunity costs of childbearing (49, 70). Concurrently, expanding accessible, high-quality public childcare services is critical to reduce the endogenous cost of childcare—a key factor inhibiting fertility identified in both our topic model and economic literature (21).

Second, implement integrated supports that reconcile work and family life, with a clear gender equity lens. Our co-word matrix revealed a strong nexus between “workplace balance” and “gender perspective,” indicating that public discourse intrinsically links fertility decisions to workplace fairness and gendered burdens. Policies must therefore combine flexible work arrangements and robust parental leave (24) with measures that actively mitigate the “motherhood penalty” in labor markets (31). Incentives for enterprises to adopt family-friendly practices should be coupled with safeguards to ensure that responsibility for care is more equally shared, fostering the conditions of the “gender revolution” associated with stabilized fertility in post-industrial societies (26, 27).

Finally, adopt a dynamic, timely policy calibration mechanism. Our findings illustrate a “temporal misalignment” (3) where rapid shifts in public sentiment outpace traditional policy cycles. To bridge this gap, policymakers should utilize frameworks like the DMPOFI developed in this study to monitor emotional inflection points in real-time. This enables interventions—such as targeted support during economic shocks or timely communication campaigns following policy announcements—to be deployed when public confidence is most fragile, thereby increasing their potential efficacy. Ultimately, moving from static, one-size-fits-all policies to a responsive, evidence-informed governance model is essential for aligning national demographic strategies with the lived realities and evolving sentiments of the public.

## 6 Conclusion

This study constructs a dynamic monitoring framework to analyze the evolution of fertility-related sentiments on social media in China from 2015 to 2024. By integrating social media text mining with empirical validation, we provide a multi-dimensional perspective that bridges the gap between “static” demographic surveys and “dynamic” public sentiment. Unlike traditional single-source studies, this approach innovatively incorporates the emotional situation in social media into the research framework, utilizing thematic models and LLMs to capture the dynamic change of social public opinion in real time.

The findings enrich the understanding of fertility intentions and reveal the impact of economic and social factors. Specifically, the trajectory of fertility intention—peaking in 2016 and declining post-2020—validates the Quantity-Quality Trade-off hypothesis in the digital age. As economic development increases the “shadow price” of childbearing (e.g., education and housing costs), policy incentives focused on “quantity” face diminishing returns against the structural demand for “quality.” Analysis based on the China General Social Survey (CGSS) further confirms that without addressing these structural costs, some policies may fail to achieve statistical significance in boosting fertility.

This study not only provides a new theoretical perspective for the study of fertility intentions but also advocates for a paradigm shift in practice. We propose transitioning from simple policy relaxation to Sentiment-Responsive Governance. Future policymakers should utilize real-time digital monitoring to identify emotional inflection points and intervene precisely when public confidence is fragile. At the same time, the combination of LLM and sentiment analysis introduced here offers a scalable solution for future public opinion analysis.

## 7 Limitation

Given the complexity of the research topic and its involvement in interdisciplinary fields, there are still some limitations in the research process. First, regarding data sources, this study used 45,810 Weibo posts crawled by Octopus, focusing solely on one social media platform. This single-platform data collection approach may limit representativeness and thus constrain the generalizability of the findings. Additionally, the reliance on publicly available social media data means that certain types of user content, particularly private or restricted posts, were excluded. This exclusion may limit the comprehensiveness of the dataset, as the views of individuals who prefer to keep their posts private are not captured. Moreover, although the study employed several classical techniques and made innovations based on them, these methods themselves also have inherent limitations. Finally, the characteristics of social media users may differ from the general population in terms of fertility intentions, which further restricts the extent to which the results reflect broader public attitudes. Building on the findings of this study, future research may extend the analytical framework in several directions. First, expanding the temporal scope could help capture longer-term dynamics in public fertility attitudes. Second, developing higher-frequency or near real-time monitoring models may enable more precise tracking of public sentiment responses to policy changes or major social events. Third, incorporating more fine-grained or multimodal data could further deepen the understanding of heterogeneity in fertility intentions and their potential behavioral implications.

## Data Availability

The original contributions presented in the study are included in the article/Supplementary material, further inquiries can be directed to the corresponding author.
