# Supplementary material for: Dynamic monitoring of public opinion on fertility intentions: based on the intersection of empirical and social media perspectives
Source: Front Public Health. 2026 Mar 19;14:1739460. doi: 10.3389/fpubh.2026.1739460 (PMC13044155; doi:10.3389/fpubh.2026.1739460)
Supplement: Supplementary file 1 [file Data_Sheet_1.pdf]

Supplementary Material

1 SUPPLEMENTARY DATA

S1 Table.

Comparison table before and after handling outliers.

S2 Table.

Comparison table for named entity identification results.

S3 Table.

Table of identification results and brief explanations.

S4 Table.

Schematic table of the effect of lexical annotation on some texts.

S5 Table.

Hierarchy of Positive Attitudes Towards Childbearing.

S6 Table.

Overall word co-occurrence matrix.

S7 Table.

Table of keywords 2015-2024.

2 SUPPLEMENTARY TABLES AND FIGURES

2.1 Figures

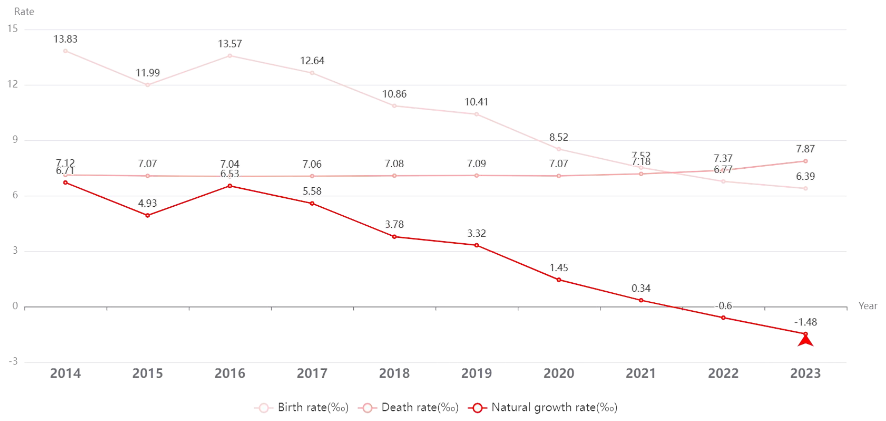

Figure S1. Birth rate,death rate and natural growth rate in the past decade (2014-2023).

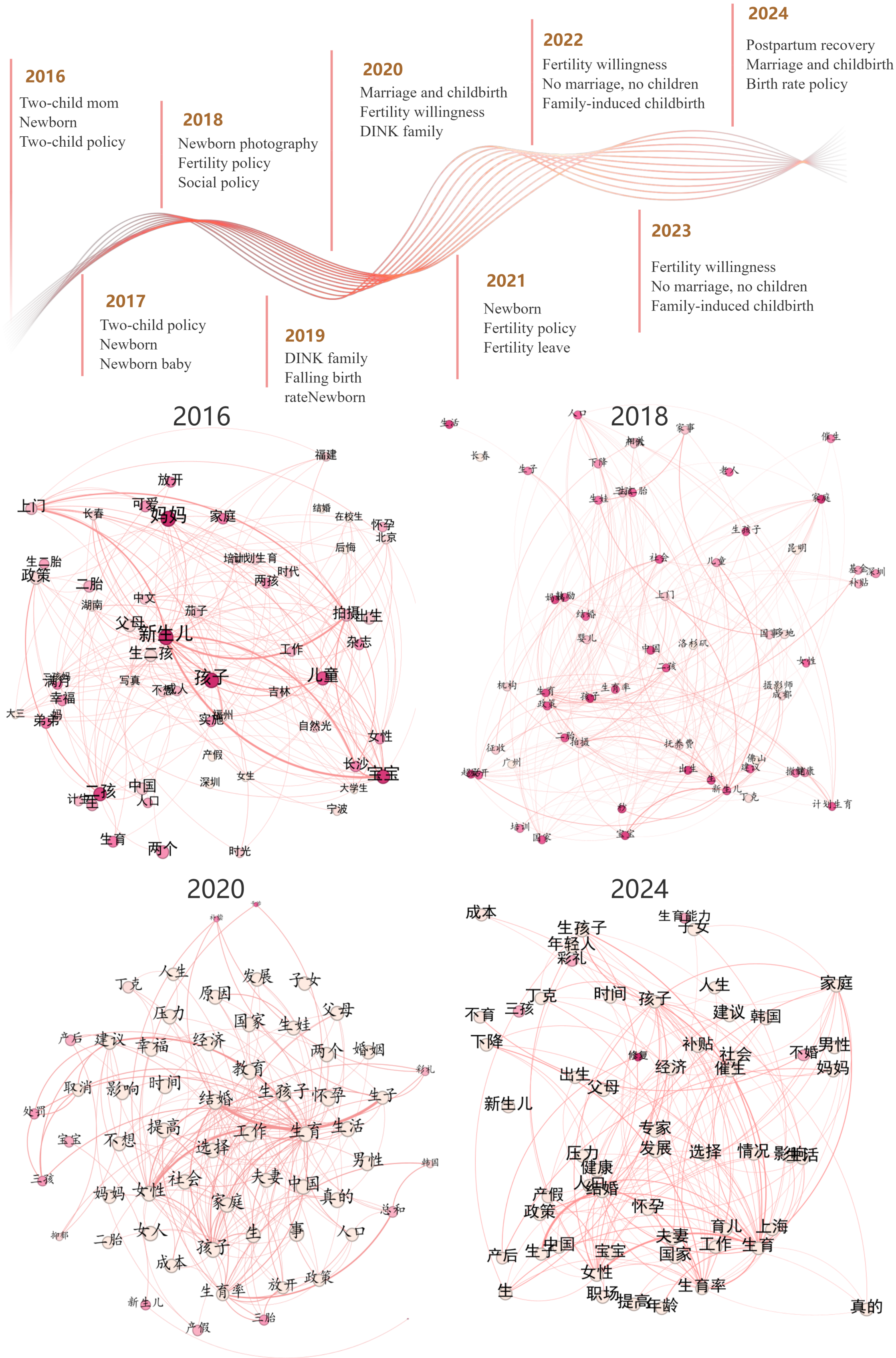

Figure S2. Change map of topic keywords from 2016 to 2024: Topics and keywords discussed from 2016 to 2024 (top half); Semantic Web structure in 2016, 2018, 2020, 2024 (bottom half).

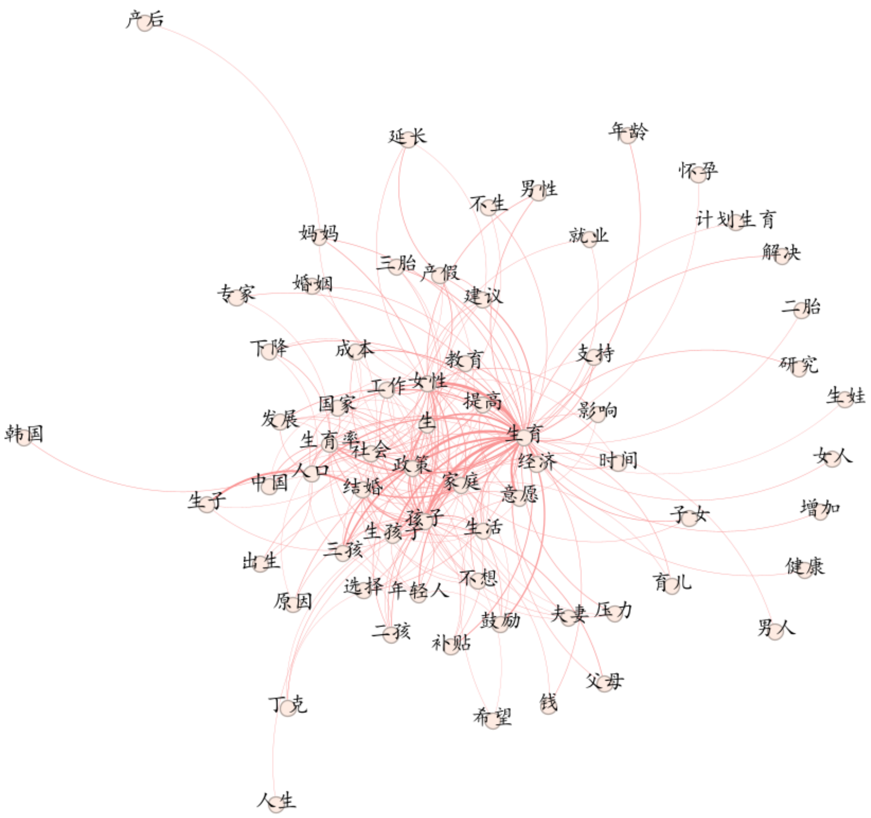

Figure S3. Overall Semantic Network Diagram.
