# Supplementary material for: Dynamic monitoring of public opinion on fertility intentions: based on the intersection of empirical and social media perspectives
Source: Front Public Health. 2026 Mar 19;14:1739460. doi: 10.3389/fpubh.2026.1739460 (PMC13044155; doi:10.3389/fpubh.2026.1739460)
Supplement: Supplementary file 2 [file Data_Sheet_2.zip › Supporting information/S2_Fig.pdf]

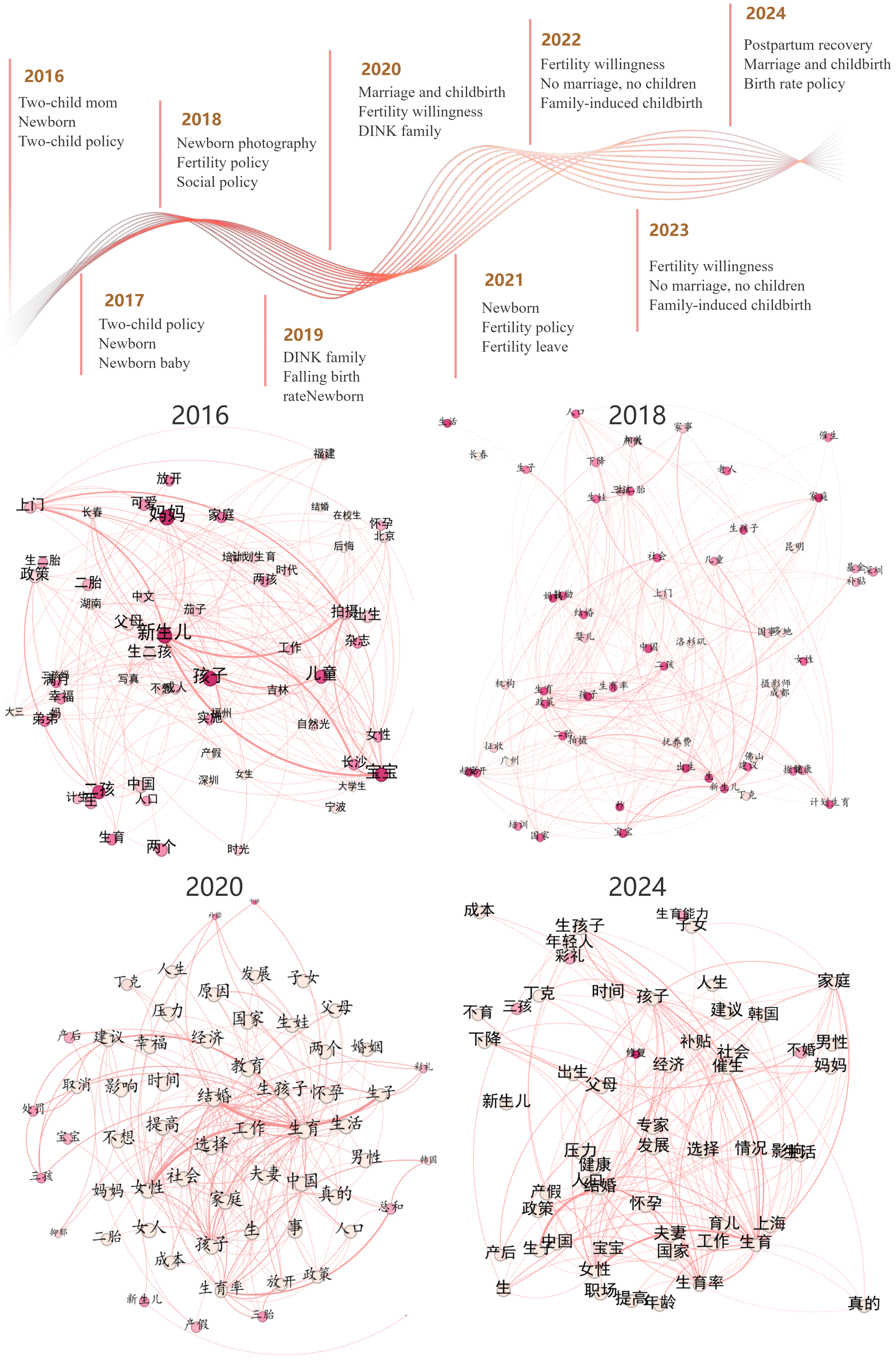
2016
Two-child mom
Newborn
Two-child policy
2017
Two-child policy
Newborn
Newborn baby
2018
Newborn photography
Fertility policy
Social policy
2019
DINK family
Falling birth
rateNewborn
2020
Marriage and childbirth
Fertility willingness
DINK family
2021
Newborn
Fertility policy
Fertility leave
2022
Fertility willingness
No marriage, no children
Family-induced childbirth
2023
Fertility willingness
No marriage, no children
Family-induced childbirth
2024
Postpartum recovery
Marriage and childbirth
Birth rate policy
2016
2018
2020
2024
