# Supplementary figures and images for: Dynamic monitoring of public opinion on fertility intentions: based on the intersection of empirical and social media perspectives
Source: Front Public Health. 2026 Mar 19;14:1739460. doi: 10.3389/fpubh.2026.1739460 (PMC13044155; doi:10.3389/fpubh.2026.1739460)

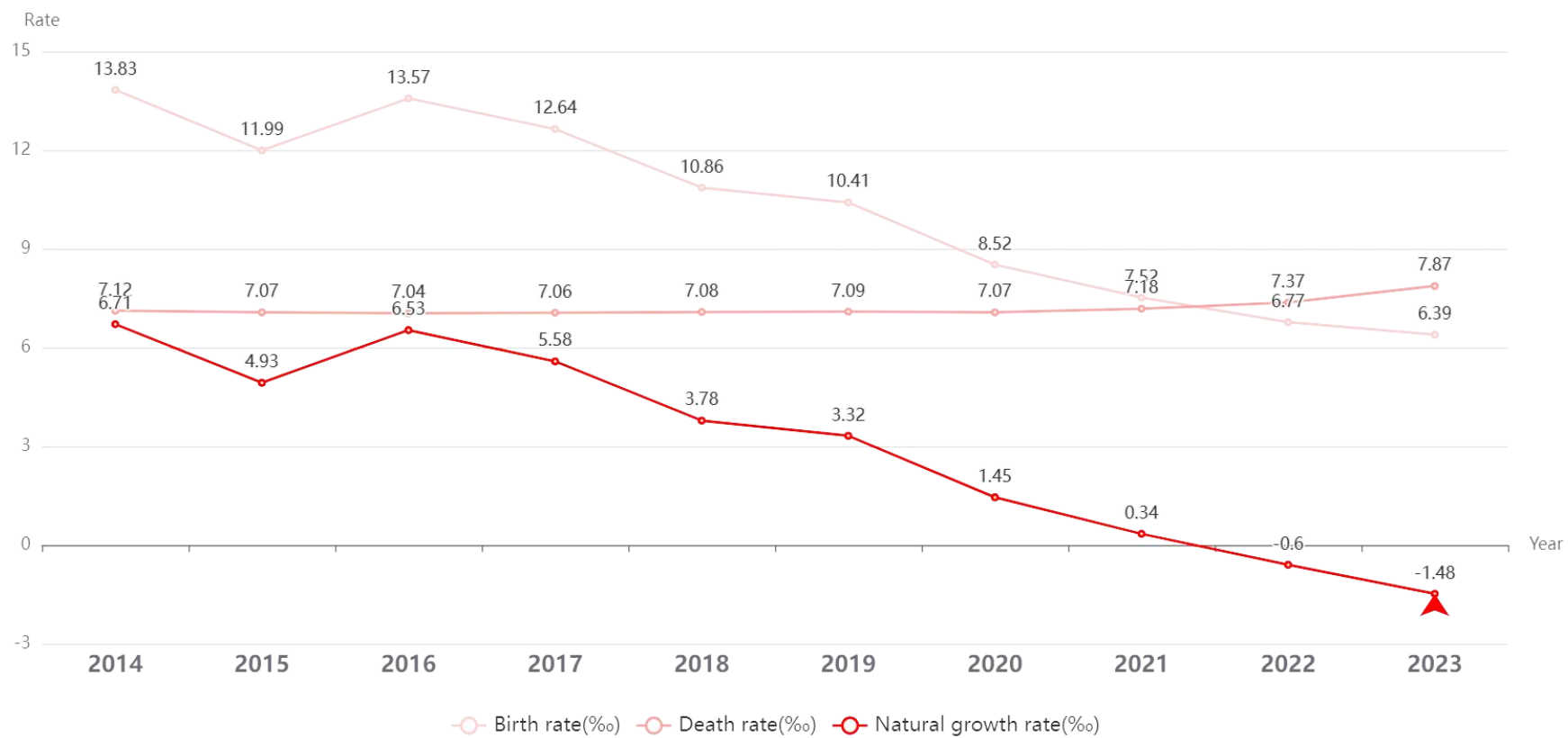

Supplement: Supplementary file 2 [file Data_Sheet_2.zip › Supporting information/S1_Fig.pdf]

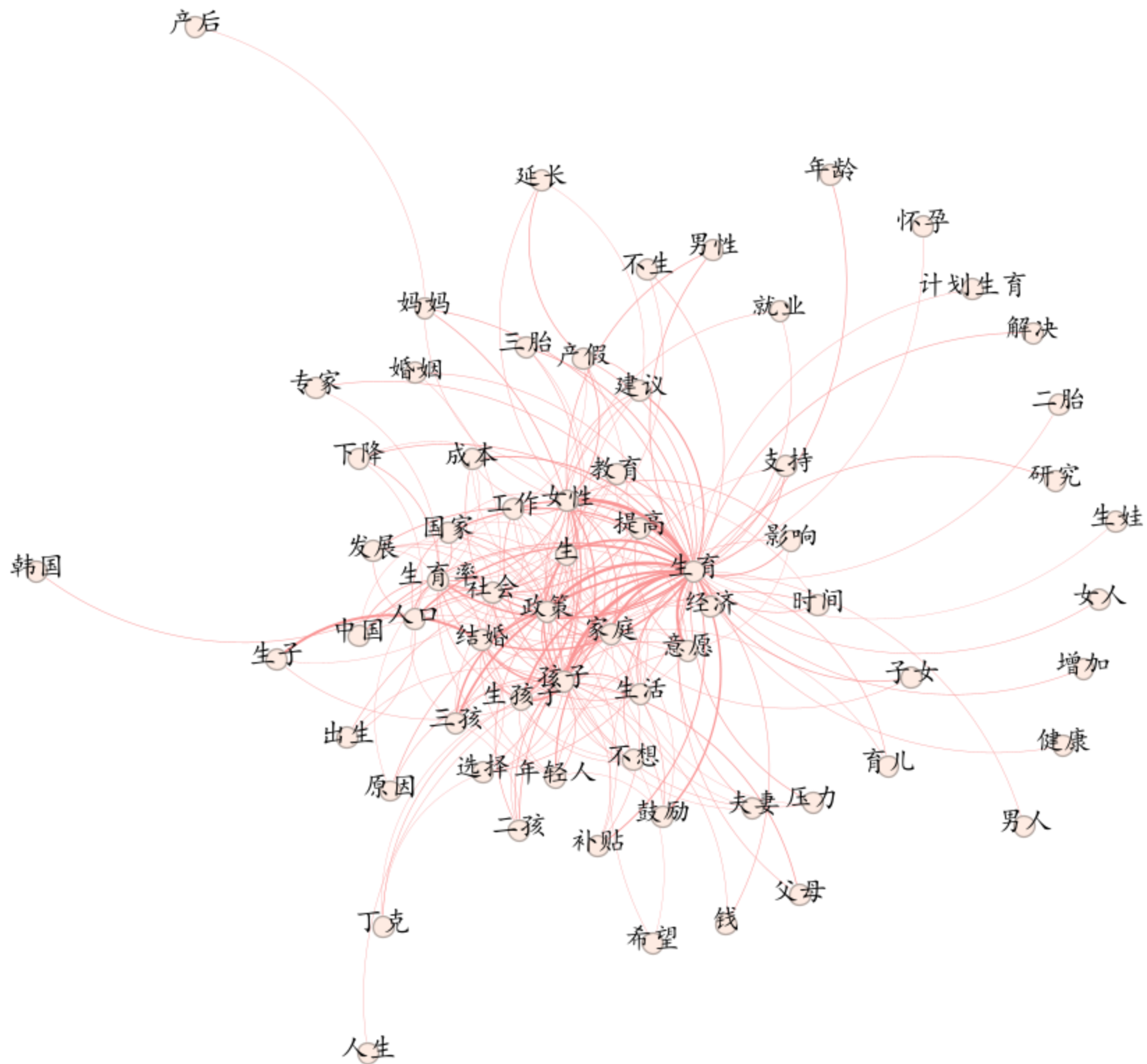

Supplement: Supplementary file 2 [file Data_Sheet_2.zip › Supporting information/S3_Fig.pdf]
